# Supplementary material for: Immunogenicity of BNT162b2 Vaccine in Patients with Inflammatory Bowel Disease on Infliximab Combination Therapy: A Multicenter Prospective Study
Source: J Clin Med. 2021 Nov 18;10(22):5362. doi: 10.3390/jcm10225362 (PMC8623980; doi:10.3390/jcm10225362)
Supplement: Supplementary file 1 [file jcm-10-05362-s001.zip › jcm-1446489-supplementary.pdf]

Supplementary Material

Table S1:Antibody response categorized by age in Infliximab combination therapy group (study group)

	Age Group			
Characteristic	Overall, N = 58[1]	Age < 35, N = 34[1]	Age >= 35, N = 24[1]	p-value[2]
**IgG BAU/mL**	99 (40, 177)	114 (60, 174)	56 (24, 177)	0.22
**IgA U/ml**	6 (3, 34)	10 (4, 37)	6 (3, 27)	0.28
**Neutralizing**	64 (23, 94)	75 (41, 94)	49 (13, 91)	0.19

[1]Median (IQR) or Frequency (%)

[2]Wilcoxon rank sum test; Fisher's exact test; Pearson's Chi-squared test

Table S2: Antibody response categorized by BMI value in Infliximab combination therapy group (study group)

	BMI levels					
Characteristic	Overall, N = 58[1]	BMI < 18.5, N = 7[1]	BMI 18.5-25, N = 26[1]	BMI 25-30, N = 16[1]	BMI >= 30, N = 9[1]	p-value[2]
**IgG BAU/mL**	99 (41, 176)	40 (20, 142)	97 (51, 180)	99 (43, 139)	143 (60, 235)	0.71
**IgA U/ml**	6 (3, 30)	4 (2, 48)	9 (4, 23)	6 (3, 27)	6 (4, 37)	0.97
**Neutralizing**	64 (28, 94)	21 (12, 85)	59 (32, 94)	63 (37, 90)	73 (61, 95)	0.65

[1]Median (IQR) or Frequency (%)

[2]Kruskal-Wallis rank sum test; Fisher's exact test

Table S3: Antibody response categorized by time-to-vaccine in Infliximab combination therapy group (study group)

		Time-to-vaccine (weeks)			
Characteristic	Overall, N = 58[1]	4-6 weeks, N = 15[1]	6-8 weeks, N = 24[1]	8-10 weeks, N = 19[1]	p-value[2]
**IgG BAU/mL**	99 (40, 177)	147 (101, 210)	93 (41, 144)	60 (19, 185)	0.10
**IgA U/ml**	6 (3, 23)	6 (3, 42)	6 (3, 19)	5 (3, 24)	0.54
**Neutralizing**	64 (23, 94)	76 (58, 96)	66 (28, 87)	48 (3, 95)	0.095

[1]Median (IQR) or Frequency (%)

[2]Kruskal-Wallis rank sum test; Pearson's Chi-squared test; Fisher's exact test
